# Draft genome sequences of clinical and environmental *Pseudomonas aeruginosa* isolates evolved under static and nutrient limitation

**DOI:** 10.1128/mra.01350-25

**Published:** 2026-04-20

**Authors:** Nahui Olin Medina-Chávez, Rhiannon E. Cecil, Ulises E. Rodriguez-Cruz, Michael Travisano, Deborah R. Yoder-Himes

**Affiliations:** 1 Ecology, Evolution and Behavior, University of Minnesota 172734 , Saint Paul, Minnesota, USA; 2 Biotechnology Institute, University of Minnesota 5635, Saint Paul, Minnesota, USA; 3 Department of Biology, University of Louisville 5170, Louisville, Kentucky, USA; 4 Department of Biology, Texas State University 7174, San Marcos, Texas, USA; 5 Minnesota Center for the Philosophy of Science, University of Minnesota 5635, Minneapolis, Minnesota, USA; Department of Biology, Queens College, Queens, New York

**Keywords:** *Pseudomonas aeruginosa*, evolution, nutrient limitation

## Abstract

We report draft genome sequences of eight *Pseudomonas aeruginosa* strains, including four ancestral strains, two clinical and two environmental, and four derivative lineages evolved over 12 weeks of static culture in minimal nutrient medium. These genomes exhibited increased pigment production (pyocyanin), altered biofilm and motility phenotypes, reflecting adaptive genomic change.

## ANNOUNCEMENT

**Fig 1:**
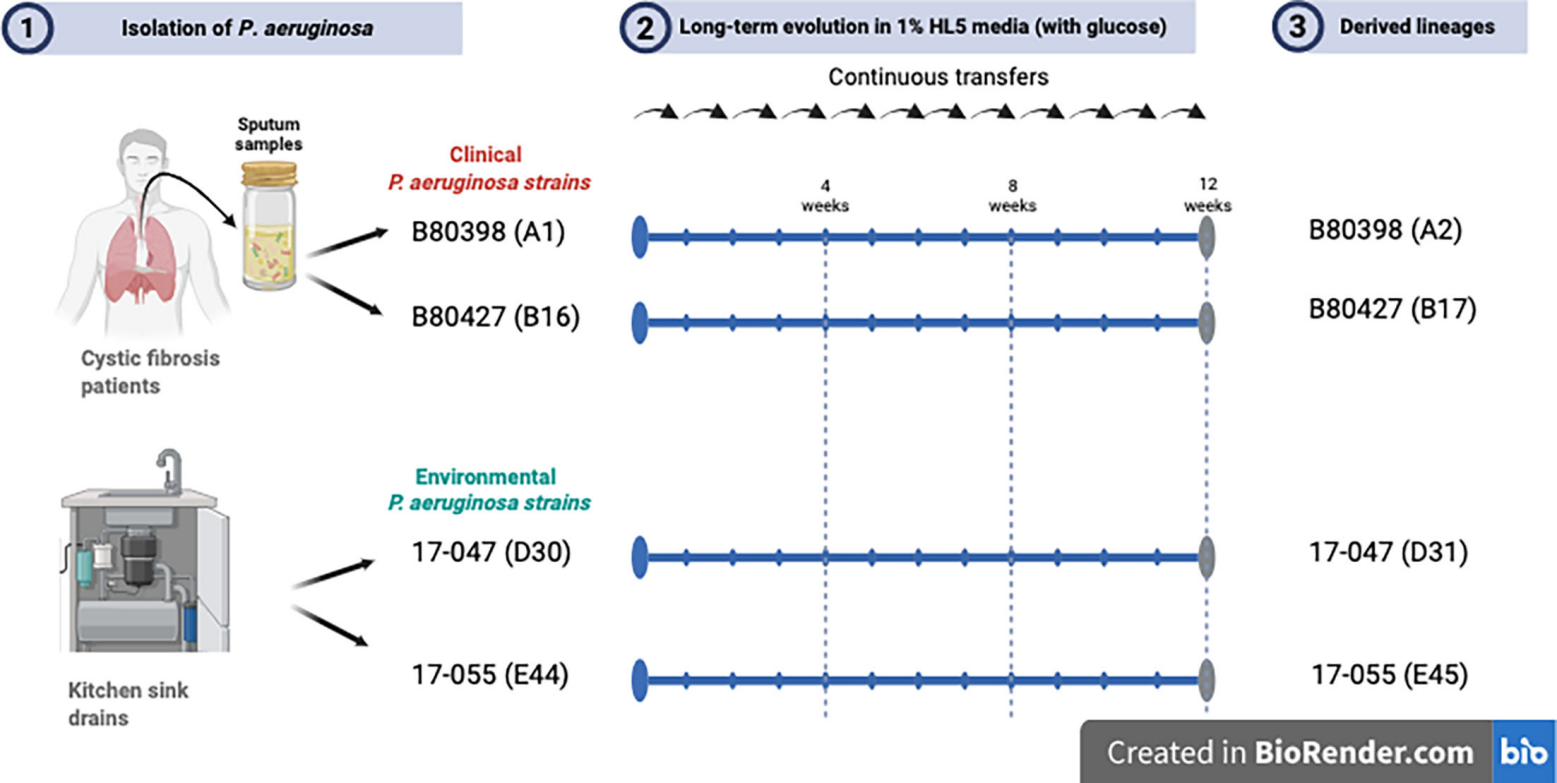
Isolation and evolution of clinical and environmental *Pseudomonas aeruginosa* strains across 12 weeks in HL5 medium.

*Pseudomonas aeruginosa* is a metabolically versatile multidrug-resistant pathogen, capable of persisting in natural and human-associated environments (1–3). Its adaptive capacity enables survival across fluctuating and nutrient-limited conditions (4). To investigate genomic adaptation under nutrient stress, we conducted a 12-week static experimental evolution study in minimal medium and sequenced ancestral and evolved isolates (Fig. 1) (2).

Four ancestral isolates, B80398 (A1), B80427 (B16), 17-047 (D30), and 17-055 (E44), along with their evolved derivatives A2, B17, D31, and E45, were obtained after 12 weeks of static culture in 1% HL5 medium (with glucose) at 22°C. Cultures were maintained with medium replacement every 2–3 days and weekly passage of surface-attached cells.

Clinical isolates (B80398 and B80427) were obtained as part of routine patient care and isolated at the Norton Hospital Microbiology Laboratory, Louisville, Kentucky, USA (38°12′55.11″ N 85°37′38.126″ W), using standard diagnostic microbiology procedures (5). The study protocol involving clinical specimens was reviewed by the institutional review board and determined to qualify for a waiver of formal review (IRB #25.0965; reference #814,003). Primary isolation was performed on LB Lennox agar plates. Species identification was conducted at the hospital microbiology core facility using mass spectrometry as part of routine clinical identification protocols (5).

Environmental isolates (17-047 and 17-055) were recovered from household sink drains sampled in the Louisville, Kentucky metropolitan area, USA. Seventy households were sampled between June 2016 and May 2017 (6). Bathroom and kitchen drains were scraped using sterile cell scrapers, and samples were collected in sterile 1× PBS and stored at 4°C prior to processing. Samples were plated on cetrimide agar and incubated at 37°C for 24–48 h. Colonies with morphology consistent with *Pseudomonas* were purified and identified using *P. aeruginosa*-specific PCR and multilocus sequence typing (6).

Genomic DNA from ancestral and evolved isolates was extracted using the Promega Wizard Genomic DNA Purification Kit. Sequencing libraries were prepared from genomic DNA fragmented by sonication (~350 bp fragments), followed by end-polishing, A-tailing, and adapter ligation. Libraries were purified using the AMPure XP system and assessed by an Agilent 5400 system before quantification by qPCR. Sequencing was performed on an Illumina NovaSeq platform (paired-end 150 bp; Novogene) to achieve ~100× coverage.

Bioinformatic pipelines were applied, using default parameters except where otherwise noted. Read quality was evaluated with FastQC v.0.20.0 (7), adapter removal and trimming were performed with Cutadapt v4.2 (8) and Trimmomatic (9), respectively. Reads were mapped to the *P. aeruginosa* UCBPP-PA14 reference genome using BWA v0.7.17 (10), sorted and processed using Samtools v1.13 (11). Variant calling detection followed GATK Best Practices using HaplotypeCaller (12), and variants were annotated using SnpEff v5.2 (13).

Draft genome assemblies were obtained with SPAdes v3.15.5 (14) and evaluated with QUAST v5.0 (15). Genome completeness was assessed using CheckM (16). Functional annotation was performed using the NCBI Prokaryotic Genome Annotation Pipeline (PGAP). Parallel SNPs occurred across evolved lineages in genes associated with phenazine biosynthesis (*phzA1/A2*), secretion systems (*vgrG*), and outer membrane transport (*oprM*), indicating convergent adaptation to nutrient stress and promoting changes in regulatory and metabolic pathways.

**TABLE 1:** Genome assembly and sequencing statistics for ancestral and selected evolved P. *aeruginosa* isolates.

Strain	Ancestral lineages				Derivative lineages			
	B80398 (A1)	B80427 (B16)	17-047 (D30)	17-055 (E44)	A2 from A1	B17 from B16	D31 from D30	E45 from E44
SRA accession	SRR31613360	SRR31613353	SRR31613338	SRR31613322	SRR31613359	SRR31613352	SRR31613336	SRR31613321
BioSample accession	SAMN53095000	SAMN53095002	SAMN53095004	SAMN53095006	SAMN53095001	SAMN53095003	SAMN53095005	SAMN53095007
Assembly accession	GCA_053452465.1	GCA_053503115.1	GCA_053452545.1	GCA_053452585.1	GCA_053452525.1	GCA_053503075.1	GCA_053452505.1	GCA_053452485.1
Total reads	11,003,760	9,610,204	9,777,748	10,048,604	10,276,000	10,847,040	18,566,944	10,879,064
Sequencing depth	187.1	169.34	162.52	175	172.79	188.48	339.24	182.51
Length (bp)	150	150	150	150	150	150	150	150
Average read quality	36	36	36	36	36	36	36	36
Mapped reads	9,766,129	8,921,569	8,676,544	8,866,855	9,119,114	10,037,516	16,760,425	9,461,172
Mapping rate %	88.75	92.83	88.74	88.24	88.74	92.54	90.27	86.97
Read length *N*_50_	150	150	150	150	150	150	150	150
*N*_50_ (kb)	1266	1266	1275	1275	1266	1266	1269	1272
Assembly coverage	217.55	198.29	194.510	370.46	203.40	222.5	211.42	195.94
Size (Mbp)	6.89	6.60	6.83	6.76	6.86	6.60	6.99	6.92
Level of completeness (%)	98.58	98.74	98.58	98.9	98.58	98.74	98.58	98.9
Largest contig (bp)	14,976	15,345	15,615	13,029	14,976	15,345	15,615	13,029
Number of contigs	6,188	5,921	6,034	6,056	6,188	5,929	6,207	6,222
GC content (%)	66.84	67.06	66.86	66.84	66.84	67.05	66.71	66.70
No. of predicted genes	6,410	6,149	6,253	6,281	6,410	6,157	6,434	6,456
CDS	5,900	5,715	5,820	5,806	5,898	5,718	5,923	5,919
tRNA	67	72	69	72	67	72	69	72
rRNA	3	4	3	4	3	4	3	4

## Data Availability

This Whole Genome Shotgun project has been deposited in DDBJ/ENA/GenBank under BioProject accession number PRJNA1194290. Individual genome and SRA accession numbers are listed in Table 1. The version described in this paper is the first submission. Raw sequencing reads are available in the NCBI Sequence Read Archive (Table 1). *P. aeruginosa* UCBPP-PA14 was used as reference genome, accession number GCA_000014625.1.
